# Sleep restriction acutely impairs glucose tolerance in rats

**DOI:** 10.14814/phy2.12839

**Published:** 2016-06-28

**Authors:** Pawan K. Jha, Ewout Foppen, Andries Kalsbeek, Etienne Challet

**Affiliations:** ^1^ Department of Endocrinology and MetabolismAcademic Medical Center (AMC)University of AmsterdamAmsterdamthe Netherlands; ^2^ Hypothalamic Integration MechanismsNetherlands Institute for NeuroscienceAmsterdamthe Netherlands; ^3^ Regulation of Circadian Clocks teamInstitute of Cellular and Integrative NeurosciencesUPR3212Centre National de la Recherche Scientifique (CNRS)University of StrasbourgStrasbourgFrance; ^4^ International Associated Laboratory LIA1061 Understanding the Neural Basis of DiurnalityCNRSFrance and the Netherlands

**Keywords:** Glucose tolerance, sleep deprivation, suprachiasmatic nucleus, type 2 diabetes

## Abstract

Chronic sleep curtailment in humans has been related to impairment of glucose metabolism. To better understand the underlying mechanisms, the purpose of the present study was to investigate the effect of acute sleep deprivation on glucose tolerance in rats. A group of rats was challenged by 4‐h sleep deprivation in the early rest period, leading to prolonged (16 h) wakefulness. Another group of rats was allowed to sleep during the first 4 h of the light period and sleep deprived in the next 4 h. During treatment, food was withdrawn to avoid a postmeal rise in plasma glucose. An intravenous glucose tolerance test (IVGTT) was performed immediately after the sleep deprivation period. Sleep deprivation at both times of the day similarly impaired glucose tolerance and reduced the early‐phase insulin responses to a glucose challenge. Basal concentrations of plasma glucose, insulin, and corticosterone remained unchanged after sleep deprivation. Throughout IVGTTs, plasma corticosterone concentrations were not different between the control and sleep‐deprived group. Together, these results demonstrate that independent of time of day and sleep pressure, short sleep deprivation during the resting phase favors glucose intolerance in rats by attenuating the first‐phase insulin response to a glucose load. In conclusion, this study highlights the acute adverse effects of only a short sleep restriction on glucose homeostasis.

## Introduction

Recent evidence convincingly shows that sleep is important for metabolic and physiological health. Results from epidemiological studies indicate that short sleep duration for a long period is correlated with obesity and type 2 diabetes (Gottlieb et al. 2005; Chaput et al. 2008; Van Cauter and Knutson 2008; Spiegel et al. 2009; Watanabe et al. 2010). For example, habitual sleep duration of <5–6 h leads to increased body mass index and impaired glucose tolerance or even type 2 diabetes (Vioque et al. 2000; Chaput et al. 2007; Watanabe et al. 2010). In addition to epidemiological studies that are mainly focused on mild chronic sleep deprivation, laboratory experiments in both human subjects and experimental animals have also linked sleep shortening with metabolic abnormalities in a more acute setting (Spiegel et al. 1999; Barf et al. 2012).

Animal experiments have shown that prolonged sleep deprivation leads to behavioral and physiological changes such as modifications in body temperature, body weight, food consumption, and energy expenditure (Rechtschaffen and Bergmann 1995; Banks and Dinges 2007; Nedeltcheva et al. 2009; Vaara et al. 2009; Barf et al. 2012; Markwald et al. 2013). Studies in humans have shown that the secretion of anabolic (growth hormone, prolactin, and testosterone) and catabolic hormones (glucocorticoids and catecholamines) may be affected by sleep disturbances (Nedeltcheva and Scheer 2014). Moreover, sleep restriction lowers plasma levels of the anorexigenic hormone leptin and elevates those of the orexigenic hormone ghrelin (Spiegel et al. 2004; Taheri et al. 2004; Barf et al. 2012). Both quality and quantity of sleep duration may affect glucose metabolism (Donga et al. 2010; Stamatakis and Punjabi 2010; Barf et al. 2012). Furthermore, a number of experimental studies with human volunteers suggest that even partial sleep disturbance leads to impaired glucose tolerance and insulin sensitivity, that is, indicators of a prediabetic condition (Spiegel et al. 1999; Tasali et al. 2008; Donga et al. 2010; Schmid et al. 2011; Robertson et al. 2013). Of note, the metabolic profile observed after sleep deprivation shares several similarities with type 2 diabetes, including decreased muscle glucose uptake, increased liver glucose output, and pancreatic *β*‐cell dysfunction (Spiegel et al. 1999; Buxton et al. 2010; Donga et al. 2010; Buxton et al., 2012).

Most of the experiments conducted in humans and animals focused on partial or complete sleep restriction during part or the whole resting period. So far no study assessed how acute, short‐term sleep deprivation affects glucose regulation. Therefore, we aimed to investigate the acute effect of short‐term sleep deprivation on glucose homeostasis in rats. In order to do so, rats were kept in a light–dark cycle and transferred to constant darkness. On the first day of constant darkness animals were subjected to an intravenous glucose tolerance test (IVGTT) immediately after a 4‐h sleep deprivation period in either the beginning or middle of the rest period.

## Methods

All the experiments were performed in accordance with the U.S. National Institute Health Guide for the Care and Use of Laboratory Animals (1996), the French National Law (implementing the European Directive 2010/63/EU), and approved by the Regional Ethical Committee of Strasbourg for Animal experimentation (CREMEAS) and the French Ministry of Higher Education and Research (#01050.01).

### Animals

Male Wistar rats (Janvier Laboratories, Le Genest‐Saint‐Isle, France) were maintained at 23°C under a 12‐h light/12‐h dark cycle (light intensity during light and dark periods [red light on] was 200 lux and <3 lux, respectively). Lights on at 07:00 am and lights off at 07:00 pm defined *zeitgeber* time (ZT) 0 and ZT12, respectively. Animals had ad libitum access to food and water and were housed individually in Plexiglas cages (28 × 28 × 40 cm) throughout the experiments. On the day of the experiment, animals were transferred into constant darkness (DD; red light, <3 lux).

### Experimental design

After a week of habituation, but only when they had reached a body weight of >300 g, animals were implanted with an intravenous silicone catheter through the right jugular vein, according to the method of Steffens (1969). Two weeks after the surgery, when animals had gained presurgery body weight again, all animals were transferred to DD. Rats (*n *= 6 per group) were either sleep deprived (SD) from circadian time (CT) 0 (defining projected time of lights on during the previous light–dark cycle) to CT4 (for early subjective day sleep deprivation) or allowed to sleep from CT0 to CT4 and sleep deprived from CT4 to CT8 (for middle of subjective day sleep deprivation) by gentle handling or left undisturbed as controls (CTR). Four hours of sleep deprivation by gentle handling is enough to enhance slow‐wave sleep during the recovery period in rats (Kostin et al. 2010). An IVGTT was performed immediately after sleep deprivation. During the final hour of sleep deprivation the jugular vein catheter was connected to a blood sampling catheter on the top of the head. This blood sampling catheter was attached to a metal collar and guided outside the animal cage. Blood sampling catheter and metal collar were kept out of reach to the rats using a counterbalanced beam. This system allowed all manipulations to be performed outside the cage without any further handling of the animals. During the experiment (including sleep deprivation and blood sampling) no food was kept in the cages. We used red headlamp during the blood sampling in DD. A glucose solution (0.5 mL, 500 mg kg^−1^ body weight) was injected as a bolus via the blood sampling and jugular vein catheter. First, a blood sample (0.2 mL) was collected (*t* = 0), immediately followed by the glucose injection. Subsequently, blood samples (0.2 mL) were taken at *t* = 5, 10, 20, 40, and 60 min. Samples were used to determine plasma concentrations of glucose, insulin, and corticosterone at these time points. The total amount of glucose in plasma and total amount of insulin released after the glucose bolus injection was calculated from the area under the curve (AUC) of every individual animal and averaged for the experimental groups.

### Laboratory method/analysis

During the experiment, blood glucose concentrations were determined by a glucometer (Accu‐Check, Roche Diagnostic, Meylan, France). Blood samples were collected in tubes on ice containing heparin and later centrifuged at +4°C. Plasma was isolated and stored at −20°C for further analysis of insulin and corticosterone. Plasma concentrations of insulin and corticosterone were measured employing radioimmunoassay kits (Millipore, Billerica for insulin and MP Biomedicals, Orangeburg for corticosterone).

### Statistical analysis

Data are presented as mean ± standard error of the mean (SEM). Statistical analysis was performed by SigmaPlot (version 12, SPSS Inc., Chicago, IL). Significance was defined at *P *< 0.05. Two‐way analysis of variance (ANOVAs) with repeated measures (rmANOVA) were performed to compare glucose, insulin, and corticosterone levels for different samples. Three‐way ANOVAs were performed to compare glucose, insulin, and corticosterone levels according to sample timing and sleep status at the two CTs. Two‐way ANOVAs were performed to compare basal glucose, insulin, corticosterone, AUCs, I/G_5‐0_, and I/G_10‐5_ between the experimental groups at the two CTs. If appropriate, post hoc analysis was performed using Tukey's test.

## Results

**Figure 1:**
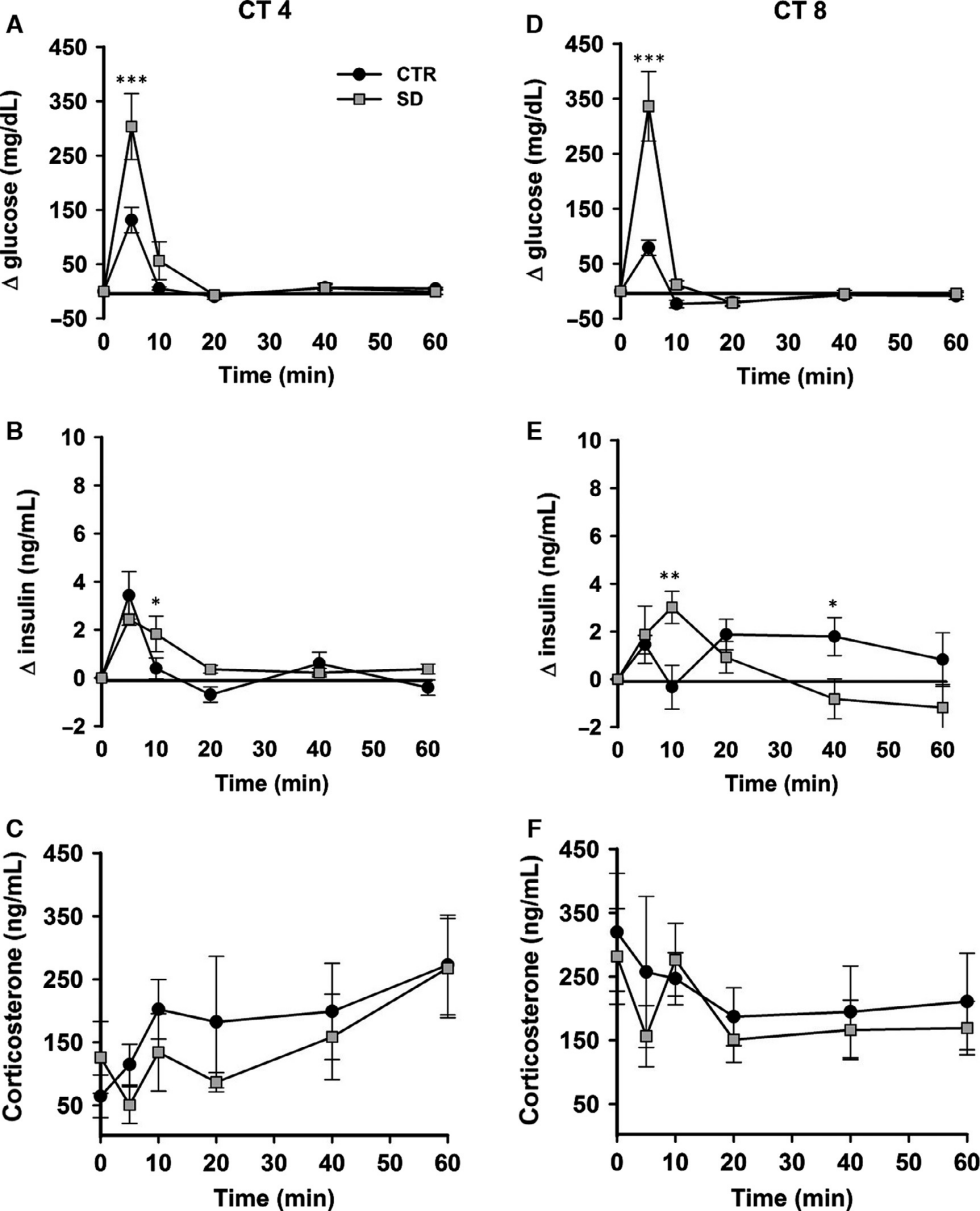
Intravenous glucose tolerance tests (IVGTTs) in rats after sleep deprivation. Relative changes in plasma glucose concentration (A, D), plasma insulin concentration (B, E), and plasma corticosterone concentration (C, F) after a glucose bolus (500 mg kg^−1^ intravenous) during IVGTTs starting at circadian time (CT) 4 and CT8. Black circles: control animals; gray squares: sleep‐deprived animals. CTR = control, SD = sleep deprived. All groups *n *= 6. Data are presented as mean ± SEM. *P < 0.05, **P < 0.005, ***P < 0.001.

Intravenous glucose tolerance test were performed immediately after the sleep deprivation (SD), during the beginning as well as in the middle of the subjective day. SD in both early and midsubjective day caused an impaired glucose tolerance. Injection of the glucose bolus resulted in an immediate and pronounced increase in plasma concentrations of glucose and insulin in both control and sleep‐deprived animals (Fig. 1A,B,D and E). Highest glucose concentrations were detected 5 min after the bolus injection, directly followed by a rapid decrease. Within 20 min after injection, glucose concentrations had returned to preinfusion concentrations again. Both during early and midsubjective day, ANOVA showed significant effects of SD (*F*_1,50 _= 6.42, *P *= 0.03 and *F*_1,50_ = 13.42, *P *= 0.004), sample timing (*F*_5,50_ = 34.85, *P *< 0.001 and *F*_5,50_ = 35.63, *P *< 0.001), and interaction (*F*_5,50_ = 5.56, *P *< 0.001 and *F*_5,50_ = 12.31, *P *< 0.001). Post hoc analysis revealed that plasma glucose levels were significantly elevated at *t*=5 min in sleep‐deprived compared to control animals at both CT4 and CT8 (*P *< 0.001). The three‐way ANOVA showed no significant effects of time of day (*P *= 0.194) or the interaction of sample timing × time of day (*P *= 0.885), SD × time of day (*P *= 0.512), or sample timing × SD × time of day (*P *= 0.587), indicating that the glucose responses at both time points were very similar.

Plasma insulin levels also increased in response to the glucose bolus in both the sleep‐deprived and control groups, at *t *= 5 min. ANOVA showed no significant effect of SD during either early or midsubjective day (*F*_1,50_ = 0.73, *P *= 0.41 and *F*_1,50_ = 0.25, *P *= 0.62), but sample timing (*F*_5,50_ = 18.72, *P *< 0.001 and *F*_5,50_ = 2.44, *P *= 0.046) and interaction (*F*_5,50_ = 2.89, *P *= 0.02 and *F*_5,50 _= 4.46, *P *= 0.002) did show significant effects at both time points. Post hoc analysis revealed that insulin levels were significantly higher in the sleep‐deprived group at *t *= 10 min during both the beginning and the middle of the rest period (*P *= 0.025 and *P *= 0.004). In addition, at *t *= 40 min plasma insulin levels were increased in the control group (CT4–8) (*P *= 0.02). The three‐way ANOVA showed significant effects of sample timing × time of day (*P *= 0.035) and sample timing × SD × time of day (*P *= 0.012), but not time of day (*P *= 0.838) or SD × time of day (*P *= 0.392) interactions, indicating small time‐course differences in insulin responses during IVGTT at CT4 and CT8 (see Fig. 1B and E).

**Figure 2:**
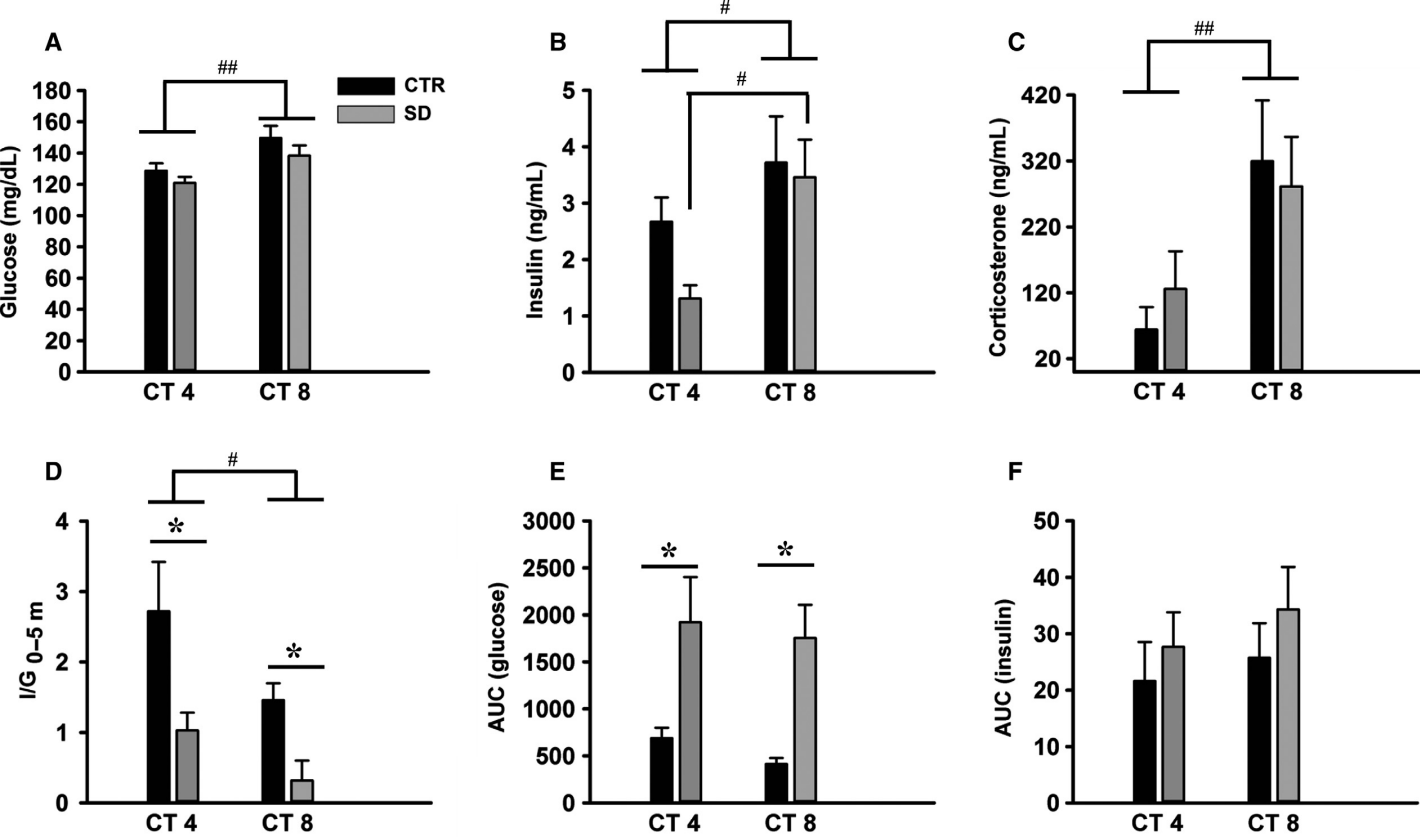
Basal glucose and hormone concentrations during intravenous glucose tolerance tests (IVGTTs) in rats after sleep deprivation (all groups *n *= 6). (A) Basal plasma glucose concentrations were significantly higher at circadian time (CT) 8 compared to CT4. (B) Basal plasma insulin concentrations were significantly higher at CT8 in the SD group. (C) Basal plasma corticosterone concentrations were significantly higher at CT8 compared to CT4. (D) I/G_t0–5_: the ratio of ∆I5‐0 to ∆G5‐0 (∆I5‐0/∆G5‐0 as a measure of the insulin response to glucose in the first 5 min) was reduced significantly after sleep deprivation in the early and late rest period. (E) AUC of the plasma glucose response and (F) the plasma insulin response after a glucose bolus at CT4 and CT8 after a 4‐h sleep deprivation. Black bars: control group; gray bars: sleep‐deprived group. CTR = control, SD = sleep deprived, AUC = area under the curve. AUC was calculated from *t* = 0 till *t* = 20 min. Data are presented as mean ± SEM. *P < 0.05 between CTR and SD; ^##^ *P *< 0.005, ^#^ *P *< 0.05 between CT4 and CT8.

To test the possibility of activation of the hypothalamo–pituitary–adrenal (HPA) axis due to sleep deprivation and intervention of IVGTTs, we measured corticosterone levels before and during IVGTTs. Basal levels of plasma corticosterone were not affected by sleep deprivation (*F*_1,20_ = 0.03, *P *= 0.8), but basal levels were higher at CT8 than at CT4 (*F*_1,20_ = 9.1, *P *= 0.007) (Fig. 2C). During IVGTTs, ANOVA showed no significant effect of SD during either early or midsubjective day (*F*_1,50_ = 0.67, *P *= 0.43 and *F*_1,50 _= 0.38, *P *= 0.54) (Fig. 1C and F). The three‐way ANOVA did not show significant effects of SD × time of day (*P *= 0.99), SD × sample timing (*P *= 0.92) and sample timing × SD × time of day (*P *= 0.9), but it detected an effect of time of day (*P *= 0.01) and sample timing *× *time of day (*P *= 0.04), indicating the higher mean corticosterone levels during the CT4‐8 IVGTT.

Basal levels of plasma glucose did not change due to sleep deprivation (*F*_1,20_ = 2.54, *P *= 0.12), though the effect of time of day on plasma glucose concentration was apparent (*F*_1,20_ = 10.33, *P *= 0.004), with higher levels later in the day (Fig. 2A). Like basal glucose, basal plasma concentrations of insulin also depended on the time of day (*F*_1,20_ = 7.55, *P *= 0.012) (Fig. 2B). Post hoc analysis showed that especially in the SD group, basal insulin was higher at CT8 compared to CT4 (*P *= 0.017).

To estimate the ability of the *β* cells to respond to a glucose challenge, we calculated insulin secretion over the first 5 min after the injection (∆I5‐0) divided by the difference between the glucose concentrations during the same time period (∆G5‐0), that is, I/G_5‐0_. ANOVA showed significant effects of *SD* (*F*_1,20_ = 12.91, *P *= 0.002) and time of day (*F*_1,20_ = 6.40, *P *= 0.02) (Fig. 2D, Table 1), with I/G_5‐0_ being lower at CT8. Post hoc analysis revealed that SD significantly decreased the I/G_5‐0_ in both early and late subjective day (*P *= 0.008 and *P *= 0.044).

**Table 1:** *β* cells responsiveness to glucose load expressed as I/G

	CT4		CT8	
	CTR	SD	CTR	SD
I/G_5‐0_	2.72 ± 0.7	1.03 ± 0.25^a^	1.46 ± 0.24	0.21 ± 0.28^a^
I/G_10‐5_	0.03 ± 0.005	−0.0002 ± 0.004^a^	0.014 ± 0.009	−0.003 ± 0.007

CTR = control, SD = sleep deprived.

Data are presented as mean ± SEM (*n *= 6/group).

a *P *< 0.05 between CTR and SD.

We further tested the ability of the *β* cells to respond to a glucose load at 10 min after injection. For this, we calculated the I/G_10‐5_. ANOVA showed significant effects of SD (*F*_1,20_ = 10.8, *P *= 0.004), but not time of day (*F*_1,20_ = 1.2, *P *= 0.27) or its interaction (*F*_1,20_ = 0.48, *P *= 0.49) (Table 1). Post hoc analysis revealed that SD significantly decreased the I/G_10‐5_ only during the early subjective day (*P *= 0.01).

We also analyzed the AUCs as estimation for the amount of glucose and insulin released after the bolus injection of glucose (Fig. 2E and F). ANOVA showed that SD significantly affected the AUC of glucose (*F*_1,20_ = 17.77, *P *< 0.001). Post hoc analysis revealed that SD significantly increased the glucose AUC in early and middle of subjective day (*P *= 0.01 and *P *= 0.006). On the other hand, insulin AUCs were not significantly affected by SD (*F*_1,20_ = 1.18, *P *= 0.29) and time of day (*F*_1,20_ = 0.64, *P *= 0.43).

## Discussion

There is increasing evidence from human and animal studies that disturbed sleep is associated with perturbations in glucose homeostasis (Spiegel et al. 1999; Barf et al. 2010). It is not clear, however, how acute sleep deprivation in terms of duration and timing during the rest period impacts on glucose metabolism. In the present study, we show in rats that a short period (4 h) of sleep deprivation is sufficient to impair glucose tolerance and reduce the early‐phase insulin response to an intravenous glucose load.

### Methodological considerations

The detrimental impact on glucose metabolism of short sleep duration over many days together with misaligned or irregular sleep has been reported in several studies (Briancon‐Marjollet et al. 2015). A few studies also investigated the effects of acute sleep restriction (i.e., within one circadian cycle) on glucose homeostasis in humans (Schmid et al. 2009; Donga et al. 2010) and rats (Barf et al. 2010). In both cases, the effects of sleep restriction were tested at only one time point. In both humans and rats glucose homeostasis is strongly influenced by time of day (Kumar Jha et al. 2015). Among others, glucose tolerance improves from the beginning of the rest period to the onset of the activity onset (la Fleur et al. 2001). Such daily variations may thus modulate the effects of sleep deprivation on glucose metabolism. Therefore, we set out to investigate whether the effects of sleep deprivation are influenced by time of day.

For sleep deprivation during early daytime, rats were forced to be awake during the first 4 h of the usual resting period (CT0–4), thus prolonging the period of wakefulness from about 12 h to 16 h. For sleep deprivation later during the light period, there are two options: either keeping the rats awake during a longer time span (e.g., 20 h) or allowing sleep during the early part of the rest period followed by sleep restriction during the latter part of the rest period. We chose the latter option as it permits to test the effect of a similar period of sleep deprivation (i.e., 4 h), but occurring at a different time of day. Notwithstanding, the fact that with sleep deprivation during the latter part of the light period sleep propensity was probably decreased compared to the rats sleep deprived in early morning, glucose tolerance was similarly altered in rats sleep deprived in either the early or middle part of the rest period. Thus, independent of time of day and sleep pressure, sleep restriction is capable of altering glucose homeostasis. In fact, the adverse effect of sleep deprivation on glucose tolerance was much stronger than the diurnal variation in glucose tolerance. Thus, the effect of sleep deprivation completely overruled the improvement of glucose tolerance during the light period as seen in the control animals.

In human studies that investigated the effects of acute sleep restriction (Schmid et al. 2009; Donga et al. 2010), lights were on during sleep restriction, which could stimulate wakefulness and inhibit melatonin secretion (Redlin 2001; Chellappa et al. 2011). In rats light exposure has been reported to stimulate glucocorticoid release (Buijs et al. 1999) and increase plasma glucose (Challet et al. 2004). Thus, to avoid interferences with the outcomes studied, lights were turned off during the present experiment. Moreover, to rule out any putative bias due to changes in food intake of sleep‐deprived rats, food was removed before the start of sleep deprivation and during blood sampling.

Several procedures have previously been used to induce sleep deprivation in rodents, including forced locomotion, gentle handling, and short platform over water. Gentle handling has the advantage to avoid the confounding effect of hyperactivity triggered by forced locomotion. In addition, it is thought to prevent the stressful effects of platform over water and forced locomotion. Our assumption that gentle handling is suitable for short periods of sleep deprivation is supported by finding similar levels of basal blood corticosterone in control and sleep‐deprived rats, indicating that the experimental groups were not stressed by gentle handling.

### Glucose tolerance and hormonal changes

In our study, a single period of 4 h of sleep deprivation either in early or middle of the light period did not modify the basal levels of plasma glucose, insulin, and corticosterone, a finding consistent with the lack of significant effect of a single night limited to 4.5 h sleep in human subjects (Schmid et al. 2009). The data from the IVGTT show that sleep deprivation in rats strongly reduces glucose tolerance, as evidenced by the rise in plasma glucose concentrations to higher levels and for a longer time. Several effects may participate in the reduced glucose tolerance. First, although the total amount of insulin released in the sleep‐deprived group was not changed (Fig. 2F), the reduced early insulin responses at both time points investigated indicates a reduced or at least inadequate sensitivity of the *β* cells. During the first 5 min after injection of the glucose bolus insulin levels were similar in the control and sleep‐deprived group, but the responsiveness of *β* cells to the glucose load was significantly reduced in the sleep‐deprived group at CT4 and CT8, and at CT4 this effect even remained present in the next 5 min. These findings are very similar to those of a previous rat study using a much longer period of sleep deprivation (i.e., 20 h) (Barf et al. 2010).

The decreased glucose tolerance in sleep‐deprived animals during IVGTT may results from either higher glucose production or less glucose uptake. The data from the present study could not differentiate whether the hyperglycemia is due to reduced glucose uptake or more glucose production. The reduced early insulin response in the sleep‐deprived groups will result both in a reduced glucose uptake as well as a lesser inhibition of glucose production. In order to understand further the mechanism of hyperglycemia, experiments using the stable isotope dilution technique to determine endogenous glucose production need to be done. Sleep deprivation might trigger glucagon release, which would subsequently result in a higher endogenous glucose production. Although at a first glance, this hypothesis appears unlikely because acute sleep deprivation has an inhibitory effect on circulating glucagon levels in humans (Schmid et al. 2009), further assays of plasma glucagon are needed to evaluate this possibility.

An alternative explanation for the increased glucose levels during the IVGTT could be an increased activity of the HPA axis, as a consequence of stress during acute sleep deprivation. In humans, most studies reported no acute change in glucocorticoid levels after sleep deprivation (Everson and Crowley 2004; Donga et al. 2010), although delayed effects (i.e., the day after) have been reported (Leproult et al. 1997). By contrast, depending on the procedure of sleep deprivation in animal studies, sleep disturbances can increase glucocorticoid release (Baud et al. 2013). However, no differences were reported in plasma corticosterone levels between sleep‐deprived and control rats (Barf et al. 2010). In the present study, basal levels of plasma corticosterone and corticosterone release during IVGTTs were not different in sleep‐deprived rats as compared to undisturbed controls, ruling out the possibility of major acute activation of the adrenal via the HPA or sympatho‐adrenal axis.

### Possible mechanisms

Our results revealed an altered insulin response to the glucose load during the first 5 min in sleep‐deprived animals. The diminished early‐phase response of insulin after sleep deprivation suggests a reduced or impaired sensitivity of the *β* cells to a glucose challenge. This defect may depend on disturbances in the sensitivity of the pancreatic *β* cells to glucose and/or its control by the autonomic nervous system. The latter possibility is supported by the fact that sleep deprivation results in sympathetic activation and release of catecholamines in the general circulation (Levy et al. 2009). Hyperactivity of the sympathetic branch of the autonomic nervous system may lead to insulin resistance (Egan 2003). Thus, the reduction in the early‐phase insulin response to glucose might be related to an increased sympathetic and/or decreased parasympathetic activity. Moreover, increased activity of the sympathetic nervous system would also stimulate glucose production. Future work should determine possible changes in the sympathovagal balance under the present conditions of sleep deprivation.

Considering that some actions of sleep deprivation on peripheral functions may result from sympathetic activation, what could be the central structures mediating these effects? A likely candidate is the hypothalamic orexin system, because this neuropeptide is involved not only in the regulation of the sleep/wake cycle, but also in the daily rhythm of glucose metabolism (Sakurai 2007; Kalsbeek et al. 2010b). Activity of orexin neurons in the perifornical region of the hypothalamus is highest during the wake period and during sleep deprivation (Estabrooke et al. 2001). These orexin neurons also participate in the control of endogenous glucose production in the liver via the autonomic nervous system (Yi et al. 2009). Furthermore, orexin appears to regulate insulin sensitivity, because mice lacking orexin show an age‐related development of systemic insulin resistance (Hara et al. 2005; Tsuneki et al. 2008). Finally, orexin has bidirectional effects on hepatic gluconeogenesis via the autonomic nervous system (Tsuneki et al. 2015). To test whether orexin neurons are involved in the autonomic control of hepatic glucose production and/or pancreatic sensitivity to glucose, orexin antagonist and organ‐specific denervation studies should be performed during sleep deprivation.

Like orexin, also the serotonin system is involved in arousal and the regulation of glucose metabolism (Asikainen et al. 1997; Versteeg et al. 2015). Injection of serotonin leads to hypoglycemia in rats and mice (Yamada et al. 1989; Sugimoto et al. 1990). Mice deficient in serotonin reuptake transporters and the 5‐HT_2c_ receptor in pro‐opiomelanocortin neurons of the arcuate nucleus in the hypothalamus show impaired glucose metabolism (Xu et al. 2010; Chen et al. 2012). The daily rhythm of SCN serotonin was shown to be severely impaired in glucose intolerant hamsters, indicating a functional link between the SCN, serotonin, and glucose metabolism (Luo et al. 1999). However, additional experiments are needed before the hypothalamic serotonin system can firmly be implicated in the sleep deprivation‐induced changes in glucose metabolism.

NPY is another hypothalamic neuropeptide involved in the control of feeding, arousal, and glucose metabolism (Szentirmai and Krueger 2006; Kalsbeek et al. 2010a; Wiater et al. 2011). Chronic sleep deprivation studies have shown increased expression of hypothalamic NPY (Koban et al. 2006; Martins et al. 2010). Central administration of NPY results in an increase in EGP in rats, probably by increasing hepatic glucose production (Kalsbeek et al. 2010a). The i.c.v. administration of NPY causes insulin resistance via activation of sympathetic output to the liver (van den Hoek et al. 2008). NPY‐containing neurons in the arcuate nucleus also project to the paraventricular nucleus of the hypothalamus (PVN), which is a relay center for the hypothalamic integration of glucose metabolism. Therefore, the presently observed impaired glucose tolerance might have been mediated through an enhanced stimulation of NPY receptors in the hypothalamus.

### Biomedical perspectives

The present study investigated the acute effects of sleep deprivation on glucose homeostasis in rats. Our data show that disturbance of the sleep–wake rhythm during early or late subjective day by short sleep deprivation acutely affects glucose metabolism by impairing glucose tolerance. Our results show that prolonged wakefulness (sleep deprivation during the early resting period) and short duration sleep deprivation (sleep deprivation in the middle of the rest period) impair glucose tolerance to the same extent.

The sleep–wake cycle is oppositely phased in nocturnal and diurnal species according to the astronomical light/dark cycle, while plasma glucose concentrations also show oppositely phased rhythms between nocturnal and diurnal rodents (Dardente et al. 2004). Therefore, it would be interesting to determine whether acute sleep deprivation during the resting period induces the same alterations of glucose metabolism in a diurnal rodent, that is, being active during the light period as are humans.

Unraveling the mechanisms that underlie the deleterious effects of sleep deprivation on glucose metabolism in rodents under tightly controlled conditions may be ultimately relevant for applications in humans.

## Conflict of Interest

None declared.
